# Ultrasound-guided removal of embedded fishbone

**DOI:** 10.1055/a-2307-5672

**Published:** 2024-05-17

**Authors:** Keerti Paida, Saurabh Gupta

**Affiliations:** 1 Gastroenterology, Concord Repatriation General Hospital, Sydney, Australia; 2 Faculty of Medicine, University of Sydney, Sydney, Australia; 3 Gastroenterology, Sydney Adventist Hospital, Sidney, Australia; 4 School of Medicine and Psychology, Australian National University, Canberra, Australia

A 60-year-old woman was referred to the emergency department after having abdominal pain for 2 weeks after eating a whole fish. Outpatient abdominal computed tomography (CT) found a 45-mm foreign body lodged in the distal stomach, with a 17 × 14 mm collection and adjacent fat stranding ( Fig. 1 ). Outpatient gastroscopy was unsuccessful in visualizing the bone.

**Fig. 1:**
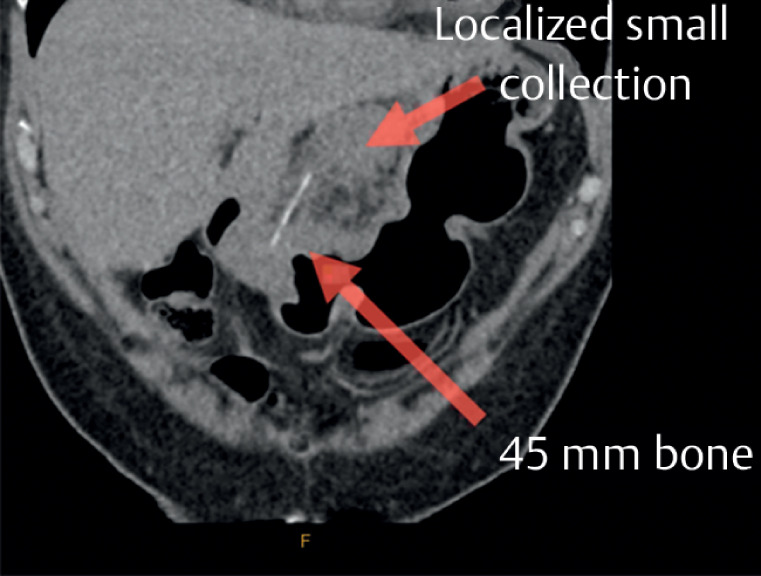
Computed tomography showing a 45-mm foreign body lodged in the distal stomach, with a 17 × 14 mm collection.

On presentation, she had mild epigastric pain but no signs of peritonitis. She was afebrile, hemodynamically stable with systemic features of infection (white cell count 5.8 × 10 ^9^ cells/L and C-reactive protein 34.4 mg/L). A surgical opinion was sought; however, it was decided to attempt endoscopic ultrasound (EUS)-guided retrieval.

Gastroscopy showed only a shallow erosion with no foreign body visible ( Fig. 2 ). Linear EUS showed the tip of the foreign body embedded beneath the mucosa, penetrating thickened muscularis and with a localized collection at the deep aspect ( Fig. 3 , Fig. 4 ). The exact site of the tip of the foreign body was marked with a tattoo using a standard fine-needle aspiration needle, and cap-fitted gastroscopy was used to improve visualization. An endoscopic retrograde cholangiopancreatography needle-knife incised the overlying mucosa at the tattoo site. Under EUS guidance, rat tooth forceps safely grasped and removed an intact fishbone ( Fig. 5 , Video 1 ). Given the localized collection, the tract was left open to allow drainage into the gastric lumen. Diet was reintroduced immediately. After 24 hours of intravenous antibiotics, the patient was discharged to complete an oral course, with no complications.

**Fig. 2:**
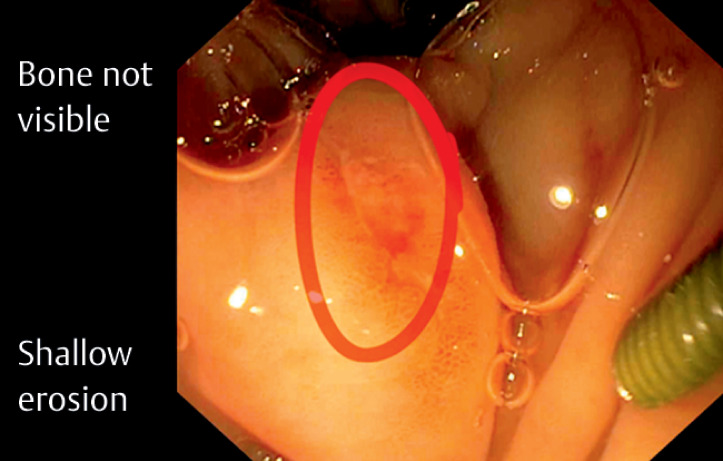
Gastroscopy showed only a shallow erosion (red circle) with no foreign body visible.

**Fig. 3:**
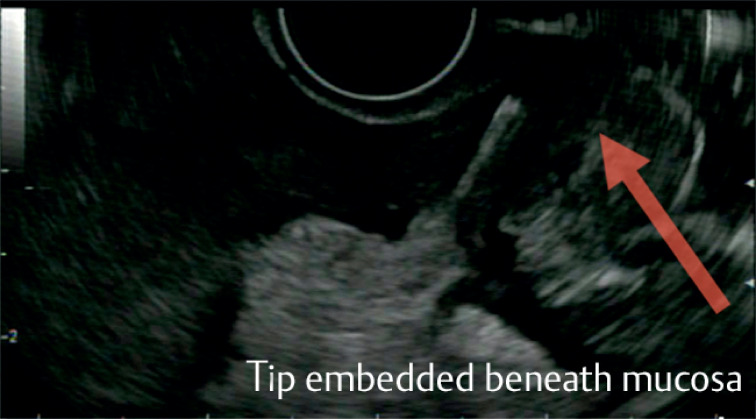
On endoscopic ultrasound, the tip of the foreign body was seen embedded beneath the mucosa.

**Fig. 4:**
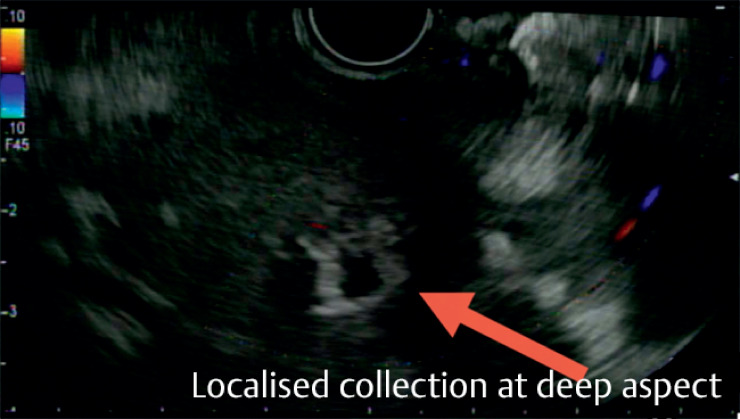
Localized collection at the deep aspect.

**Fig. 5:**
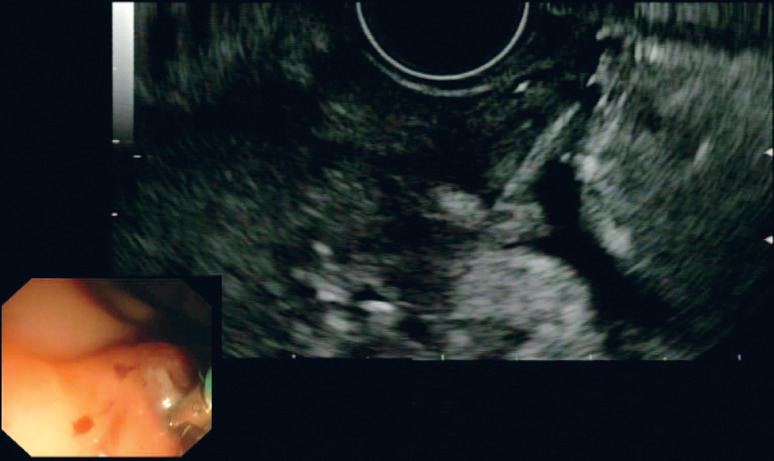
Rat tooth forceps grasped the foreign body under ultrasound guidance.

Endoscopy showed erosion, but the foreign body was not visible. The site was marked with a tattoo. The operator used rat tooth forceps under ultrasound guidance to grasp and successfully retrieve the foreign body.Video 1

The prognosis for ingested foreign bodies is quite good, with most passing within 24 hours without intervention 1 . In cases where the foreign body has possibly been embedded for some time, CT findings can guide the choice of laparoscopic vs. endoscopic approach 2 . Risk factors associated with complications (e.g. perforation, bleeding, abscess formation) are longer duration of impaction (>24 hours) and bone length (>3 cm) 3 . This case had both risk factors; however, as the foreign body had only embedded just under the mucosal surface, EUS-guided retrieval was successful in retrieving the foreign body.

Endoscopy_UCTN_Code_TTT_1AS_2AB

## Notes

Endoscopy E-Videos https://eref.thieme.de/e-videos
*E-Videos* is an open access online section of the journal *Endoscopy* , reporting on interesting cases and new techniques in gastroenterological endoscopy. All papers include a high-quality video and are published with a Creative Commons CC-BY license. Endoscopy E-Videos qualify for HINARI discounts and waivers and eligibility is automatically checked during the submission process. We grant 100% waivers to articles whose corresponding authors are based in Group A countries and 50% waivers to those who are based in Group B countries as classified by Research4Life (see: https://www.research4life.org/access/eligibility/ ). This section has its own submission website at https://mc.manuscriptcentral.com/e-videos .
